# Divulging the Critical Role of HuR in Pancreatic Cancer as a Therapeutic Target and a Means to Overcome Chemoresistance

**DOI:** 10.3390/cancers13184634

**Published:** 2021-09-15

**Authors:** Dimitrios Goutas, Nikolaos Goutas, Stamatios Theocharis

**Affiliations:** 1 First Department of Pathology, Medical School, National and Kapodistrian University of Athens, 11527 Athens, Greece; stamtheo@med.uoa.gr; 2 Department of Forensic Medicine and Toxicology, Pathology, Medical School, National and Kapodistrian University of Athens, 11527 Athens, Greece; ngoutas@med.uoa.gr

**Keywords:** HuR, pancreatic adenocarcinoma, pathogenesis, treatment, chemoresistance, tumor microenvironment

## Abstract

**Simple Summary:**

With pancreatic cancer incidence constantly rising, constituting one of the most lethal type of cancers worldwide, the need for discovering novel therapeutic targets and approaches becomes of the utmost importance. Meanwhile, modern eating habits, hyperadiposity, mutational burden affecting core signaling pathways and the unique tumor microenvironment of pancreatic cancer tissues intermingle and form a disease that is lethal and hard to treat. The importance of HuR in pancreatic cancer has repeatedly been observed and represents a key molecule in pancreatic carcinogenesis and chemoresistance. Therefore, creating and obtaining new therapeutic skills against HuR protein could prove to be the answer to pancreatic cancer therapy.

**Abstract:**

Pancreatic cancer is set to become the most lethal and common type of cancer worldwide. This is partly attributed to the mutational burden that affects core signaling pathways and the crosstalk of tumor cells with their surrounding microenvironment, but it is also due to modern eating habits. Hyperadiposity along with the constant rise in metabolic syndrome’s incidence contribute to a state of metaflammation that impacts immune cells and causes them to shift towards an immunosuppressive phenotype that, ultimately, allows tumor cells to evade immune control. Unfortunately, among the conventional therapeutic modalities and the novel therapeutic agents introduced, pancreatic cancer still holds one of the lowest response rates to therapy. Human antigen R (HuR), an RNA binding protein (RBP), has been repeatedly found to be implicated in pancreatic carcinogenesis and chemotherapy resistance through the posttranscriptional binding and regulation of mRNA target genes. Additionally, its overexpression has been linked to adverse clinical outcomes, in terms of tumor grade, stage, lymph node status and metastasis. These properties suggest the prospective role that HuR’s therapeutic targeting can play in facilitating pancreatic neoplasia and could provide the means to overcome chemoresistance.

## 1. Introduction

Pancreatic ductal adenocarcinoma (PDA) represents the fourth (4th) most common cause of cancer mortality in developed countries [1], with geographical variations and lifestyle factors shaping the context of its incidence. Contrary to the other cancer entities, pancreatic cancer has been linked to many risk factors involved in several different pathways, including hereditary and genetic factors [1]. Unfortunately, the vast majority of pancreatic adenocarcinomas have already spread beyond the pancreatic parenchyma at the time of diagnosis, mostly extending into the ampulla of Vater, the duodenum and the intrapancreatic portion of the common bile duct, as well as into the peripancreatic or retroperitoneal adipose tissue [2]. Several tumor suppressor genes have been linked to pancreatic neoplasia, such as *Kirsten rat sarcoma (KRAS), cyclin dependent kinase 4A (CDK4A), tumor suppressor protein 53 (TP53)* and *the SMAD family member 4 (SMAD4)* [3,4]. More commonly, *SMAD4* seems to be inactivated in 55% of pancreatic cancers, either by homozygous deletion or by loss of one allele coupled with an intragenic mutation in the second allele [5]. These mutations lead to the dysregulation of core signaling pathways, affecting both the proliferation and migration of tumor cells and also the crosstalk with their surrounding tumor microenvironment (TME) [6]. A large genomic analysis study performed by Bailey et al. [7] in 2016 of 456 PDAs led to the identification of 32 recurrently mutated genes that ultimately resulted in classifying these tumors into four subtypes, each of them correlated with specific histopathologic characteristics. Namely, these subtypes include (a) squamous; (b) pancreatic progenitor; (c) immunogenic and (d) aberrantly differentiated endocrine exocrine (ADEX) [7]. Based on the World Health of Organization (WHO), PDA is classified according to histology into colloid carcinoma (CC), signet-ring cell carcinoma (SRCC), undifferentiated carcinoma with osteoclast-like giant cells (UCOGC), adenosquamous carcinoma (ASC) and hepatoid, rhabdoid and medullary carcinoma [8]. To date, PDA prognosis remains dismal, with an average survival of 3–5 months for untreated patients and 10–20 months for patients undergoing surgical resection [9,10]; adjuvant chemotherapy with gemcitabine or 5-fluorouracil (5-FU) prolongs survival only slightly [11]. The failure of “all-comer” treatment approach, along with the molecular diversity and the unique tumor microenvironment of PDA [12,13,14], makes the turn towards precision medicine more paramount than ever. Advances in genomic-driven personalized medicine have shown promise in identifying unique therapeutic targets in individual patients, personalizing treatment selection. However, the ability of PDA cancer cells to compensate through different signaling pathways, as well as the fact that various genetic lesions are found to be pivotal for tumor progression, constitutes a challenge for this approach. HuR, an RNA binding protein, participates in posttranscriptional control of RNAs, such as splicing, polyadenylation, mRNA stabilization, localization and translation [15]. Additionally, the HuR molecule controls gene expression in multiple areas of malignant transformation, thus regulating the expression of multiple cancer-related genes. Its protein levels and localization have been linked to pathologic inflammation, malignant transformation and cancer progression by evading immune destruction, inducing angiogenesis and promoting tumor-associated inflammation [16,17,18].

**Figure 1:**
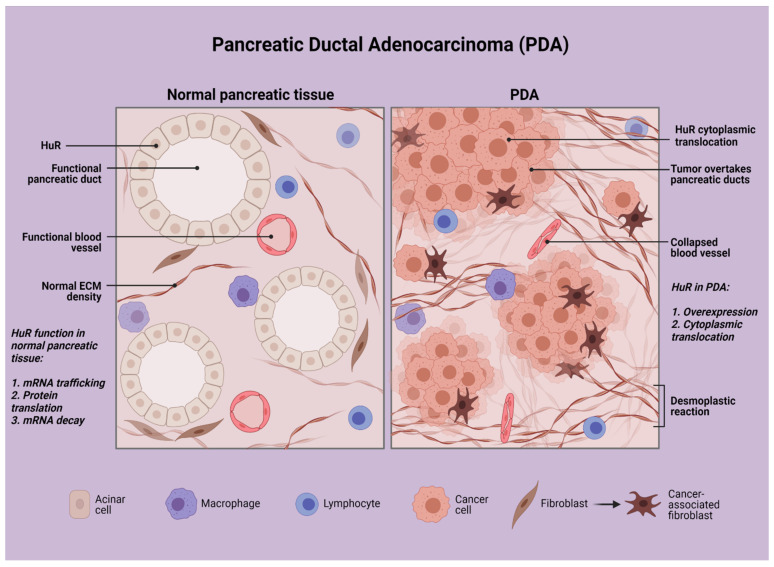
Schematic representation of pancreatic ductal adenocarcinoma in contrast with normal pancreatic tissue. Created with BioRender.

Taking the above into consideration, one should consider a target that can be stimulated by the unique tumor microenvironment of PDA cancer cells (Figure 1), a target that can be found in abundance in cancer cells, while not in pancreatic normal cells, and a target that can offer a significant survival advantage in PDA cancer cells. In this review, we focus on HuR, an RNA-binding protein, and its therapeutic significance in pancreatic neoplasia and inflammation.

## 2. HuR and Neoplasia

HuR is an RBP encoded by the *ELAVL1* gene that acts by stabilizing mRNAs and regulating gene expression [19]. HuR is composed of various structural motifs, such as RNA recognition motif (RRM), dsRNA binding domain, zinc fingers and others. RBPs have crucial roles in various cellular processes, such as cellular function, transport and localization. Posttranscriptional control of RNAs, such as splicing, polyadenylation, mRNA stabilization, localization and translation are some of the major functions displayed by RBPs [15]. HuR encodes a 32 kD protein composed of three RNA-binding domains that belong to the RRM family. More specifically, it is RRM-1 and RRM-2 that are responsible for AU rich elements (ARE) binding, and it is RRM-3 that binds to the mRNA poly(A) tail [20]. The expression of various molecules is regulated by HuR protein through different posttranscriptional mechanisms, including mRNA trafficking, protein translation and mRNA decay. Furthermore, HuR can control gene expression in multiple areas of malignant transformation, regulating multiple cancer related genes’ expression. HuR overexpression has been repeatedly linked to malignant transformation, and increased nuclear/cytoplasmic HuR expression has been associated with patients’ prognosis in different malignancies [21]. The above-mentioned properties advocate for the adverse clinical outcomes related to HuR protein overexpression in various cancer types, including advanced stage, positive lymph nodes and poor survival [22,23,24,25,26,27].

It has been demonstrated that posttranscriptional gene regulation allows PDA and colorectal tumor cells to survive under cancer-associated stress conditions [28,29,30,31,32,33]. More specifically, RBPs and micro-RNAs (miRNAs) contribute to posttranscriptional gene regulation and can directly influence gene expression by modulating mRNA stability [34,35]. Among the many RBPs and miRNAs that repress translation and foster mRNA decay, HuR represents the most potent and promising agent against cancer-associated mRNA degradation. Constantino et al. [29] studied the consequences of adjusting HuR levels in pancreatic cancer cells, and they found out that the cells overexpressing HuR in their cytoplasm were highly more sensitive to gemcitabine. More specifically, in pancreatic cancer cells, deoxycytidine kinase (dCK) mRNA interacts with HuR encoding the enzyme that metabolizes and activates gemcitabine [29]. Gemcitabine activation enhances the association of HuR with dCK mRNA and consequently increases HuR’s cytoplasmic expression. Appropriately, HuR’s overexpression leads to dCK mRNA increase in pancreatic cancer cells, while its silencing reduces dCK levels. Additionally, a clinical trial performed by Constantino et al. on gemcitabine efficacy demonstrated that patients with low HuR cytoplasmic expression levels had a 7-fold increased mortality risk when compared with patients with elevated HuR cytoplasmic levels [29]. Their results enhance the belief of HuRs’ paramount role as a key mediator of gemcitabine efficacy in PDA through the posttranscriptional regulation of dCK mRNA levels.

Additionally, the harsh TME of PDAs favors the growth of the most aggressive and suitable PDA cells. As PDA tumors are set in a hypoxic, nutrient-deprived microenvironment, only the most appropriate and aggressive clonal populations will survive and thrive [36,37]. These populations are also the most resistant against the cytotoxic chemotherapeutic agents used in PDAs [4,37,38]. Consequently, PDA cells, in order to survive under these hypoxic conditions, put together a multifaceted response and activate hypoxia inducible factors (HIF), such as the proviral integration site for Moloney murine leukemia virus 1 (*PIM1*) and HuR [39,40]. Furthermore, HuR protects PDA cells under nutrient-deprived conditions by regulating the key metabolic enzyme isocitrate dehydrogenase 1 (*IDH1*) [41] and translocating it into the cytoplasm [42]. It is via the regulation of these cellular reprogramming events that HuR activates various pathways for regulating angiogenesis, intracellular pH, DNA repair, cell survival, cell motility and mitochondrial function [42,43,44].

## 3. Elevated HuR Expression Causes a Pancreatitis-Like Inflammatory Microenvironment

The role of local and systemic inflammation has been shown to be imperishably linked to PDA growth, development and metastasis [45]. The inflammatory and cancer cells of a tumor’s microenvironment are capable of producing and releasing a variety of anti- and proinflammatory cytokines and regulating the balance among them through their constant interactions [46]. The cytokines more commonly implicated in pancreatic cancer are the anti-inflammatory cytokines tumor growth factor β (TGF-β) and interleukin (IL)-10 and the pro-inflammatory IL-6 and IL-1β. IL10 and tumor necrosis factor-α (TNF-α) play a paramount role in the microenvironment of PDA tumors [46]. Low-grade chronic inflammation in systemic diseases, such as metaflammation in patients with metabolic syndrome or diabetes mellitus (DM), enhance the risk of cancer, particularly pancreatic cancer [47]. This subclinical chronic inflammation linked with hyperadiposity or DM enhances the belief that there is a link between chronic inflammation and pancreatic cancer [47]. More specifically, obese patients demonstrate an augmented release of the pro-inflammatory adipokine leptin and a reduced release of the anti-inflammatory adipokine adiponectin, while at the same time, a shift from the M2 to the M1 macrophages infiltrating the adipose tissue triggers the release of pro-inflammatory cytokines, predominately TNF-α and IL-6 [48]. Nevertheless, in addition to the role of metaflammation in PDA in patients with metabolic syndrome, the consequences of pathways linked to nutrition and the gut microbiome also seem to contribute significantly to pancreatic neoplasia [49,50]. Excessive eating exerts a solid immunomodulatory effect by giving rise to a subclinical inflammatory status with pro-inflammatory cytokines and trophic hormones and by impacting immune cells through the gut microbiota, which can ultimately lead to a shift towards an immunosuppressive phenotype that allows tumor cells to escape immune control [48].

HuR’s role in inflammation is now long established [21,51]. HuR regulates mRNAs responsible for encoding proinflammatory proteins (TNF-α, IL-6, COX-2) but also proteins that block the production of anti-inflammatory factors (thrombomodulin). More specifically, HuR has been implicated in several diseases for augmenting inflammation, including asthma, inflammatory bowel disease and rheumatoid arthritis, among others [52]. Peng et al. [16] studied the consequences of HuR overexpression in a transgenic mouse model that had a >2-fold elevation of pancreatic HuR expression. Histological examination revealed an intense fibroinflammatory reaction, along with a marked increase in inflammatory infiltrates, fibrosis, ductal complexes and acinar atrophy—features suggestive of chronic pancreatitis [16]. Moreover, immunohistochemical examination showed an increased expression of TNFα, cyclooxygenase-2 (COX-2), vimentin, α-smooth muscle actin (α-SMA) and collagen-1 [16]. Additionally, cluster of differentiation (CD) [45], CD3, CD86 and IL-6 were increased in the transgenic mouse model in comparison with the mice without HuR overexpression, further outlining the fibroinflammatory response commenced by HuR protein. Additionally, Peng et al. attempted to correlate HuR’s expression with tumorigenesis by examining hematoxylin and eosin (H/E) sections from pancreata of TC mice older than 10 months old, without, however, any precancerous pancreatic intraepithelial neoplasia (PanIN) lesions or PDA being observed; this led to the conclusion that in the absence of any known driver of gene mutations, HuR overexpression alone does not initiate tumorigenesis [16]. Nevertheless, when combining *KRAS* mutant mice with HuR overexpression compared with only *KRAS* mutant mice, they observed an increased incidence of PanIN lesions and up to 5-fold increased incidence of PDAs in the mice overexpressing HuR, supporting the notion that the inflammatory microenvironment induced by HuR expression in *KRAS* mutant mice promotes tumor formation [16]. These findings directly incriminate HuR as a promoter of pancreatic cancer, predominantly within the context of inflammation.

## 4. Agents Interacting with HuR Expression and How HuR’s Inhibition Could Affect Tumor Progression

**Figure 2:**
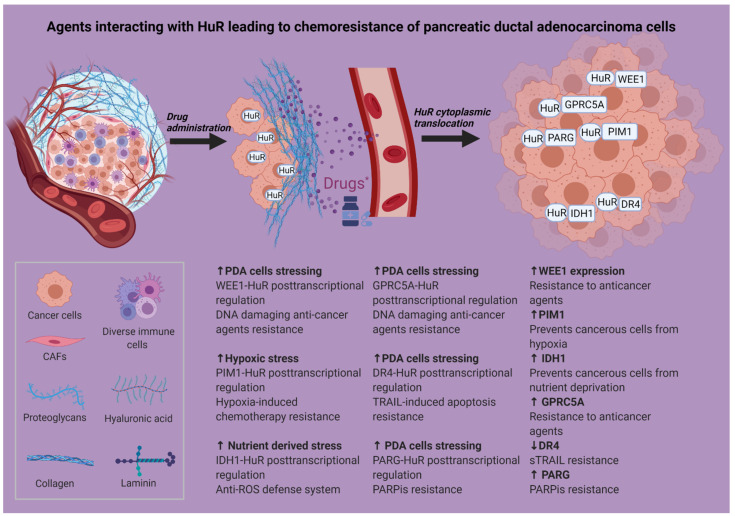
Agents interacting with HuR causing chemoresistance of pancreatic ductal adenocarcinoma cells. Created with BioRender. Abbreviations in Figure 2: CAFs: cancer-associated fibroblasts; DR4: death receptor 4; ECM: extracellular matrix; GPRC5A: G protein-coupled receptor class C group 5 member A; HuR: human antigen R; IDH1: isocitrate dehydrogenase; PARG: poly(ADP-ribose) glycohydrolase; PARP: poly (ADP-ribose) polymerase; PARPis: poly (ADP-ribose) polymerase inhibitors; PIM1: proviral integration site for Moloney murine leukemia virus-1; ROS: reactive oxygen species; sTRAIL: soluble TRAIL; TRAIL: TNF-related apoptosis-inducing ligand; HuR: human antigen R. Created with BioRender. * Gemcitabine, 5-fluorouracil, oxaliplatin.

Many different strategies have been used in an attempt to modify or suppress HuR’s action in cancer [27], including inhibiting its cytoplasmic translocation, decreasing its expression via siRNAs or inhibiting its binding to target mRNAs. On top of that, intense research has been performed to obtain data regarding the synergistic use of HuR inhibition with chemotherapeutic and a variety of other agents [53,54,55,56,57] (Figure 2, Table 1).

### 4.1. Synergistic Use of HuR Inhibitors with Abemaciclib

Dhir et al. [53] analyzed the effect of combining abemaciclib with HuR inhibition by using two validated inhibitors, CMLD-2 and pyrvinium pamoate, in PDA cell lines [53]. The result was that both cell lines demonstrated a decreased number of colonies compared with monotherapy. Furthermore, PDA cell lines transfected with siHuR oligonucleotides, revealed decreased IC_50_ rates to abemaciclib in comparison with si-negative cells. This highlights the increased sensitivity that PDA cell lines obtained from the mentioned therapeutic combination regimen and the potential that this combination entails.

### 4.2. Agents Interacting with HuR Inducing Chemoresistance

PIM1 represents a hypoxia-inducing, pro-oncogenic, serine-threonine kinase that only recently turned out to be a key regulator of hypoxia-induced chemotherapy resistance in PDAs. The molecular mechanism underlying its overexpression in pancreatic carcinomas is based on the presence of cis-acting AREs in the PIM1’s mRNA 3′ untranslated region, which mediates an interaction with HuR in a tumor hypoxia context [41]. More specifically, HuR, in response to hypoxic stress, translocates from the nucleus to the cytoplasm of PDA cells and stabilizes PIM1 mRNA transcript, causing PIM1 protein overexpression. The HuR-mediated PIM1 protein overexpression prevents cancerous cells from hypoxia through phosphorylation and inactivation of BAD (Bcl-2-associated death promoter) and activation of MEK 1/2 (mitogen activated protein kinase kinase) [41]. Selective inhibition of HuR by MS-444 blocks its homodimerization and its cytoplasmic translocation, therefore rendering the PDA cells susceptible to oxaliplatin and 5-FU [41]. These results elucidate the role of HuR and its prosurvival properties in PDA and provide evidence that its selective inhibition and disruption of PIM1 regulation could be the key to interrupting this chemotherapeutic resistance mechanism.

IDH1 is a NADPH (nicotinamide adenine dinucleotide phosphate)-generating enzyme that has been demonstrated to be posttranscriptionally stabilized by HuR; via this posttranscriptional regulation, HuR manages an anti-ROS (reactive oxygen species) defense system. It is well known that HuR protects PDA cells not just from hypoxia but also from nutrient-derived stress [39,41]. A study conducted by Zarei et al. showed that, under nutrient-deprived conditions, PDA cells were less sensitive to gemcitabine in PDA xenografts in hypoglycemic mice, compared with the hyperglycemic mice [42]. Similar results were observed in a retrospective study of patients with elevated serum glucose levels treated with gemcitabine, as they revealed an improved OS (overall survival). Furthermore, Zarei et al. [42] identified an enhanced antioxidant defense as a driver of chemoresistance. More precisely, ROS levels were increased in vitro either by nutrient deprivation or gemcitabine treatment, but withdrawing nutrients from PDA cells before gemcitabine treatment enhanced this effect [42]. However, HuR expression reduced ROS levels under low glucose, whereas HuR silencing augmented ROS levels. Investigation via CRISPR and RNAi (RNA interference) of the factor responsible for maintaining survival of PDA cancer cells under nutrient-deprived conditions revealed HuR to be the implicated agent. Importantly, studies in HuR-null PDA cell lines demonstrated IDH1 as the single HuR-regulated antioxidant enzyme [42]. These findings support the notion that selective inhibition of HuR could break the HuR-IDH1 regulatory axis and serve as a promising therapeutic target.

Lal et al. [32] demonstrated that stressing PDA cancer cells with DNA damaging anti-cancer agents (carboplatin, cisplatin, oxaliplatin, mitomycin C and PARP-inhibitors) resulted in HuR’s translocation from the nucleus to the cytoplasm [32]. What is even more interesting, is that HuR knockdown in PDA cells resulted in their sensitization to the above agents, while HuR’s overexpression led to resistance. HuR was implicated with DNA-damaging anti-cancer agents by the acute upregulation of WEE1. Actually, WEE1, a mitotic inhibitor kinase, participates in the regulation of the DNA damage repair pathway, and its therapeutic inhibition along with chemotherapy is currently under clinical trials investigation for cancer treatment [58,59,60,61,62,63,64,65]. Furthermore, Lal et al. demonstrated the role of WEE1 as a HuR target, both in vitro and in vivo, by revealing the direct binding of HuR to WEE1 mRNA and that HuR small interfering RNA (siRNA) knockdown and/or overexpression affects the WEE1 protein levels, especially following DNA damage. HuR stimulation of WEE1 subsequently leads to an increase in the γH2AX levels, causes Cdk1 phosphorylation and facilitates cell cycle arrest at the G2/M transition. Additionally, they demonstrated a novel acute checkpoint mechanism that involves WEE1 and by which cells can block and potentially withstand any sudden DNA damage insult experienced [32]. Taking these parameters into consideration, targeting the HuR-WEE1 interactions could be a promising novel approach towards patients receiving chemotherapy and, thereby, enhancing their therapy’s outcomes. Generally, therapies focusing on translocating targets, such as HuR, and its targeting mRNAs, such as WEE1, could turn out to be more efficient therapeutic strategies than the targeting of PDA cells’ genetic alterations [32].

GPRC5A (G protein-coupled receptor class C group 5 member A) is a protein coding gene that demonstrates a dual behavior—acting as an oncogene in certain cancers and as a tumor suppressor in other cancers [66]. Zhou et al. [54] attempted to establish the impact of GPRC5A overexpression in PDA cell lines and provided an association between its overexpression and HuR’s role in pancreatic cancer. After exhibiting that GPRC5A mRNA levels hold the second highest average expression among different cancer types in pancreatic cancer, they examined and compared its expression levels in normal pancreatic tissues, primary PDAs and metastatic tumors. As a result, GPRC5A was shown to be overexpressed in primary and metastatic PDA tumors, and even more in the metastatic sites [54]. This was further validated through immunohistochemical analysis. In an attempt to analyze the posttranscriptional regulation of GPRC5A, the authors performed luciferase assays, demonstrating that HuR binds to at least one site in the 3′-UTR of GPRC5A. More specifically, after the cellular stress caused by gemcitabine treatment, HuR translocates to the cytoplasm where it binds with the GPRC5A mRNA and induces a monotonous increase in GPRC5A protein levels for at least 18 h. After this, HuR and GPRC5A mRNA association returns to background levels and its posttranscriptional control decreases [54]. Additionally, GPRC5A levels were increased after treatment both with 5-FU and oxaliplatin suggesting that other factors could also be involved in regulating GPRC5A expression in response to chemical stressors. These interactions of GPRC5A with HuR, gemcitabine and the other chemotherapeutic agents imply a potential pro-oncogenic role for this gene; therefore, targeting of these interactions could augment the death rate of pancreatic cancer cells post-chemotherapy treatment.

TRAIL (TNF-related apoptosis-inducing ligand) is a type II transmembrane protein harvesting an important role in cancer onset, progression and apoptosis [67]. TRAIL directly induces apoptosis by engaging cell surface death receptors (DR) DR4 and DR5, constituting a possible molecular target in cancer therapeutics. It has been previously demonstrated that HuR and DR5 expression share an inverse relation in vitro and in PDA patient tissues. Additionally, HuR is capable of binding to DR5 mRNA and suppressing its protein expression, leading to a decrease in apoptosis [68]. Nevertheless, due to the fact that DR4, and not DR5, has been proven to be a more potent trigger for TRAIL-induced apoptosis in PDA cells [69], Romeo et al. studied the effects of HuR levels and their correlation with DR4 expression levels and TRAIL resistance in PDA [55]. In order to obtain information regarding the association of DR4 and DR5 expression with PDA cell sensitivity to soluble (s)TRAIL treatment, the authors compared the IC_50_ for every cell line from the sTRAIL killing curve, revealing a strong direct correlation among DR4 cell surface expression and TRAIL sensitivity, compared with the association observed among DR5 expression and TRAIL sensitivity [55]. They further demonstrated that HuR not only translocates to the cytoplasm in response to sTRAIL treatment but also that HuR has the ability to posttranscriptionally bind DR4 mRNA through the 3′-UTR. Additionally, they utilized specific siRNA to silence HuR in PDA cell lines in the presence of sTRAIL, which resulted in an increase in DR4 cell surface protein expression, suggesting that HuR plays a role in the downregulation of TRAIL-induced DR4 mRNA expression [55]. Therefore, strategies focused on decreasing HuR cytoplasmic concentration in PDA patients could enhance the efficacy of certain treatment regimens, such as TRAIL.

Poly (ADP-ribose) polymerase (PARP) is a family of proteins involved in a number of cellular processes, such as DNA repair, genomic stability and programmed cell death [70]. Although PARP inhibitors (PARPi) initially showed promising results, it turned out that most tumors would develop drug resistance [71,72]. Chand et al. [73] demonstrated that the antitumor response to PARPi in PDA is largely controlled by the HuR-dependent stabilization of poly (ADP-ribose) glycohydrolase (PARG) [73]. More specifically, they attempted to assess the role of HuR in PARPi response in PDA cell lines via HuR’s knockout. As a result, CRISPR knockout of HuR ensued a 20-fold increase in sensitivity to several PARPis (olaparib and veliparib) [73]. Furthermore, they demonstrated that PARPis induced a cytoplasmic HuR translocation, which, however, could be blocked using a small molecule inhibitor, MS-444, that would prevent HuR dimerization. Additionally, HuR inhibition with MS-444 resulted in a significant decrease in PARG expression and an associated accumulation of total polyADP-ribosylation (PARylation) [73]. Taking these data together, inhibition of HuR inhibits PARG overexpression and function and could possibly be utilized to enhance the efficacy of PARPi.

Considering, the aforementioned, properties of HuR and the effects that it is capable of exerting both in tumor progression and in therapy induction, it automatically raises the probability of becoming a potential drug target. Inhibiting HuR could assist in overcoming the major issue of chemoresistance that PDA cancer patients encounter, because to date, no matter of the protocol used, PDA entails one of the most dismal prognoses among cancers.

## 5. Clinical Trials

Studies performed exhibiting the role of HuR as a biomarker both in monotherapy and in drug combination regimens are limited and have restricted outcomes [31,73]. McAllister et al. utilized tissue microarrays (TMAs) from the Radiation Therapy Oncology Group (RTOG) 9704 trial [31] and attempted to evaluate the relationship between HuR and dCK in patients receiving 5-FU. In contrast with previous studies displaying an association between HuR expression and prolonged OS in patients undergoing gemcitabine therapy, no significant association could be observed among patients with increased or decreased tumor’s HuR cytoplasmic expression and disease-free survival (DFS). However, for the first time, a close correlation was seen between HuR expression and dCK levels (increased HuR cytoplasmic levels followed a dCK increased expression), most probably attributed to the posttranscriptional regulation that dCK is subjected to. Furthermore, they observed that HuR and dCK were not associated with patients’ OS, which came as a discordance with previous studies demonstrating that stabilization of dCK transcript by HuR enhanced gemcitabine efficacy and metabolism [29,31,73]. These findings were largely attributed to the use of radiation in the patients of the RTOG 9704 trial, which resulted in interference with HuR’s biological behavior [74]. More specifically, preexisting molecular data revealed that ionizing radiation induces uncoupling of the mRNAs bound to HuR, inhibiting the posttranscriptional regulation of HuR’s target genes [74]. In order to validate this, McAllister et al. [31] tried initially to examine the importance of sequencing order of gemcitabine and radiation on pancreatic cancer cells survival. As a result, they observed that there was 70% more pancreatic cancer cell death when gemcitabine preceded radiation treatment, as compared with the opposite pattern [31]. Additionally, removing HuR with siRNAs exhibited no difference in OS, regardless of the chemoradiation protocol used. Regarding dCK expression, the greatest amount was observed in the protocol where radiation preceded gemcitabine treatment. This was demonstrated in ribonucleoprotein-immunoprecipitation assays, where when radiation was initially administered there was an 80% reduction in dCK mRNA bound to HuR [74]. Additionally, they observed that PDA cells treated with 5-FU demonstrated a HuR cytoplasmic translocation. Nevertheless, and regardless of the fact that HuR levels were not predictive of OS, neither in patients receiving gemcitabine nor in those receiving 5-FU, the data shown above are, generally, compatible with a treatment approach where gemcitabine treatment heralds radiation, leading to an augmentation of pancreatic cancer cell killing.

As one could argue the effects that radiation displays in regard to HuR’s biological behavior, Tatarian et al. [73] utilized tumor samples from the international European Study Group of Pancreatic Cancer-3 trial, where patients with PDA received either gemcitabine or 5-FU adjuvant monotherapy in the absence of adjuvant radiation, to provide evidence of HuR’s role as a prognostic and predictive biomarker [73]. Unfortunately, cytoplasmic HuR (cHuR) expression was not indicative of DFS or OS in the total cohort. Nevertheless, in the 20% of the patients in the study who demonstrated an increased cHuR, a significant survival advantage was observed in those receiving 5-FU. On the contrary, these patients’ tumors were fairly resistant to GEM. Meanwhile, patients with low cHuR expression did not display any difference in median DFS, regardless of treatment arm. Nevertheless, despite the acknowledged limitations in their study, Tatarian et al. provided enough evidence to support the notion that cHuR may be predictive of 5-FU efficacy in patients with resectable PDA and high cHuR, thereby challenging previous studies that highlight HuR as promoter of chemotherapy resistance [32,41,42]. It is probable that HuR coordinates certain transcripts that play a major role in 5-FU sensitivity, or that high cHuR levels could be a marker of rapid cell division, and these cells could be especially susceptible to 5-FU therapy.

## 6. Conclusions

Overall, it has been shown that the rough tumor microenvironment of PDA cancer cells renders them chemoresistant, and in combination with HuR’s posttranscriptional regulation of specific target genes (COX-2, VEGF, c-Myc, WEE1 and PIM1) [32,41,75,76,77], cell growth and survival are enhanced. These findings underline the potential role of HuR as a therapeutic target in pancreatic cancer and suggest the grounds on which PDA cancer cells often show a diminished response to chemotherapy. The molecular mechanisms of HuR protein regulation could be useful in the identification of novel targets for drug design. These could include either inhibition of HuR protein translation by using siRNAs or suppression of its cytoplasmic translocation and inhibition of HuR mRNA target binding by using small molecules that act on the ligand receptors and on intracellular proteins critical for tumor growth. The currently evolving strategy of targeting non-mutated genes or pathways, finds HuR to be a promising drug target in pancreatic cancer and other tumor types. HuR protein inhibition has occasionally been found to be more efficient when combined with other chemotherapeutic agents, especially with platinum-based drugs [78]. However, Constantino et al. [29] demonstrated in PDA cells that HuR closely associates with dCK mRNA, which in turn encodes the enzyme responsible for the metabolism and activation of gemcitabine. In a clinical correlate study, they found a 7-fold increased mortality risk in patients with decreased HuR cytoplasmic levels compared with patients with increased HuR levels [29]. Their findings not only highlight the paramount role of HuR as a key mediator of gemcitabine efficacy in PDA cells but also reveal the extreme diversity that exists among different cancer types and how this directly influences the therapeutic strategy followed. Further studies need to be conducted in order to shed light on the many questions that need answering, including the identification of the most appropriate HuR mRNA targets in pancreatic cancer, the efficacy of HuR inhibition as a monotherapy or in combination with chemotherapy and the careful investigation of its mechanism of action, as blocking HuR (e.g., in combination with gemcitabine) could probably have an adverse result in PDA patients. As HuR represents a gene regulatory protein, its role in medicine and in pancreatic neoplasia could alter the use of conventional therapies and provide new insights into the current treatment strategies.

## Figures and Tables

**Table 1:** Agents interacting with HuR in a posttranscriptional level and their therapeutic outcome.

Agent	Function	Expression	HuR Action	Mechanism	Outcome	Ref.
PIM1	Serine-threonine kinase	↑	Cytoplasmic translocation Binds PIM1 mRNA	Phosphorylation and inactivation of BAD Activation of MEK 1/2	Prevents cancerous cells from hypoxia	[41]
IDH1	NADPH generating enzyme	↑	Cytoplasmic translocation Binds IDH1 mRNA	HuR impacts antioxidant defense by regulating IDH1	Prevents cancerous cells from nutrient deprivation	[42]
WEE1	Mitotic inhibitor kinase	↑	Cytoplasmic translocation Interacts with WEE1 mRNA	CDK1-phosphorylation Increase in the γH2AX levels Cell cycle arrest at the G2/M transition	Resistance to anti-cancer agents	[32]
Abemaciclib	Chemotherapeutic agent	---	HuR inhibition	CMLD-2 and pyrvinium pamoate	Decreased IC_50_ rates to abemaciclib Increased sensitivity of PDA cells to abemaciclib	[53]
GPRC5A	Protein binding gene Dual behavior (oncogene or tumor suppressor)	↑	HuR cytoplasmic translocation Binds GPRC5A mRNA	Posttranscriptional regulation	Monotonous increase in GPRC5A protein levels	[54]
TRAIL	Type II transmembrane protein	↓	HuR cytoplasmic translocation Binds DR4 mRNA	Posttranscriptional regulation	Downregulation of TRAIL-induced DR4 mRNA expression Suppresses apoptosis	[55]
PARPis	Inhibitors of PARP (family of proteins involved in several cellular processes)	---	Cytoplasmic translocation Binds PARG mRNA	HuR dependent stabilization of PARG	PARPis resistance	[57]

Abbreviations: HuR: human antigen R; IDH1: isocitrate dehydrogenase; PIM1: proviral integration site for Moloney murine leukemia virus-1; GPRC5A: G protein-coupled receptor class C group 5 member A; DR4: death receptor 4; PARG: poly(ADP-ribose) glycohydrolase; PARP: poly (ADP-ribose) polymerase; PARPis: poly (ADP-ribose) polymerase inhibitors; TRAIL: TNF-related apoptosis-inducing ligand,; CDK1: cyclin dependent kinase 1; γH2AX: phosphorylated H2A histone family member X.
